# Synthesis and Cytotoxic Activity of Some Novel *N*-Pyridinyl-2-(6-phenylimidazo[2,1-*b*]thiazol-3-yl)acetamide Derivatives

**DOI:** 10.3390/molecules17044703

**Published:** 2012-04-23

**Authors:** Huaiwei Ding, Zhe Chen, Cunlong Zhang, Tian Xin, Yini Wang, Hongrui Song, Yuyang Jiang, Yuzong Chen, Yongnan Xu, Chunyan Tan

**Affiliations:** 1 School of Pharmaceutical Engineering, Shenyang Pharmaceutical University, Shenyang 110016, China; Email: dinghuaiwei627@163.com (H.D.); hongruisong@163.com (H.S.); 2 The Key Laboratory of Chemical Biology, Guangdong Province, Graduate School at Shenzhen, Tsinghua University, Shenzhen 518055, China; Email: czseasky@yahoo.com.cn (Z.C.); zhcunl@126.com (C.Z.); xintian0904@163.com (T.X.); yini0403@163.com (Y.W.); jiangyy@sz.tsinghua.edu.cn (Y.J.); 3 Bioinformatics and Drug Design Group, Department of Computational Science, National University of Singapore, Blk SOC1, Level 7, 3 Science Drive 2, Singapore 117543, Singapore; Email: csccyz@nus.edu.sg

**Keywords:** imidazo[2,1-*b*]thiazoles, cytotoxic activity, synthesis

## Abstract

A series of novel compounds bearing imidazo[2,1-*b*]thiazole scaffolds were designed and synthesized based on the optimization of the virtual screening hit compound *N*-(6-morpholinopyridin-3-yl)-2-(6-phenylimidazo[2,1-b]thiazol-3-yl)acetamide (**5a**), and tested for their cytotoxicity against human cancer cell lines, including HepG2 and MDA-MB-231. The results indicated that the compound 2-(6-(4-chlorophenyl)imidazo[2,1-b]thiazol-3-yl)-N-(6-(4-(4-methoxybenzyl)piperazin-1-yl)pyridin-3-yl)acetamide (**5l**), with slightly higher inhibition on VEGFR2 than **5a** (5.72% and 3.76% inhibitory rate at 20 μM, respectively), was a potential inhibitor against MDA-MB-231 (IC_50_ = 1.4 μM) compared with sorafenib (IC_50_ = 5.2 μM), and showed more selectivity against MDA-MB-231 than HepG2 cell line (IC_50_ = 22.6 μM).

## 1. Introduction

During the last few decades, anticancer therapy has made significantly progress, especially after the approval of a few small-molecule inhibitors, but the management of malignancies in humans is still one major concern. Extensive interest is being focused on exploring newly functional scaffolds capable of being further developed as anticancer agents. Recently, considerable attention has been paid to the design and the biological activity of compounds bearing imidazo[2,1-*b*]thiazole scaffolds due to their broad spectrum of pharmacological activities, such as antifungal [1,2,3], antibacterial [3,4,5,6] anti-inflammatory [7] and antihypertensive properties [8], as well as being used as cystic fibrosis transmembrane conductance regulator (CFTR)-selective potentiators [9]. In particular, many imidazo[2,1-*b*]thiazole derivatives have been reported to display potential antitumor activities against a variety of human cancer cell lines [10,11,12,13,14,15,16]. The recent studies demonstrated that pyrimidinyl-substituted imidazo[2,1-*b*]thiazole derivatives could inhibit Raf kinases [17], and appropriately substituted imidazo[2,1-*b*]thiazoles could be developed as dual inhibitors of IGF-IR (insulin-like growth factor receptor) and EGFR (epidermal growth factor receptor) [18] or P38 kinase inhibitors [19]. However, there is no report about imidazo[2,1-*b*]thiazole derivatives as vascular endothelial growth factor receptor (VEGFR) inhibitors. These observations elicited much interest in this fused ring system and prompted us to attempt to develop VEGFR inhibitors possessing the imidazo[2,1-*b*]thiazole core.

**Figure 1:**
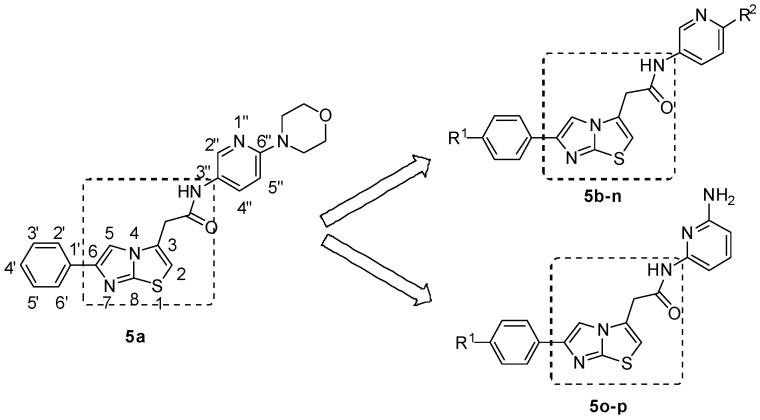
Structures of hit compound **5a** and modification position of target compounds.

Angiogenesis, the formation of new blood vessels from pre-existing ones, plays a central role in the process of tumor growth and metastasis [20]. Blockade of VEGF/VEGFR signaling pathways, which regulates proliferation and migration of endothelial cells, has been explored as a highly successful clinical strategy in cancer treatment [21]. Several VEGFR inhibitors have been approved and many are under development in clinic. Based on our interest in searching for novel biologically active molecules as VEGFR inhibitors and consideration of the potent bioactivities of compounds that possess an imidazo[2,1-*b*]thiazole scaffold, we searched Ambinter and ChemSpider chemical database using a SVM (support vector machine) screening model [22,23], and found one hit compound **5a** (ChemSpider ID: 11102329) (Figure 1) bearing an imidazo[2,1-*b*]thiazole core with potential anticancer activity. As follow-up to this observation, we have now designed and synthesized a series of novel imidazo[2,1-*b*]thiazole acetamide derivatives and assessed their *in vitro* activities.

## 2. Results and Discussion

### 2.1. Chemistry

**Scheme 1:**
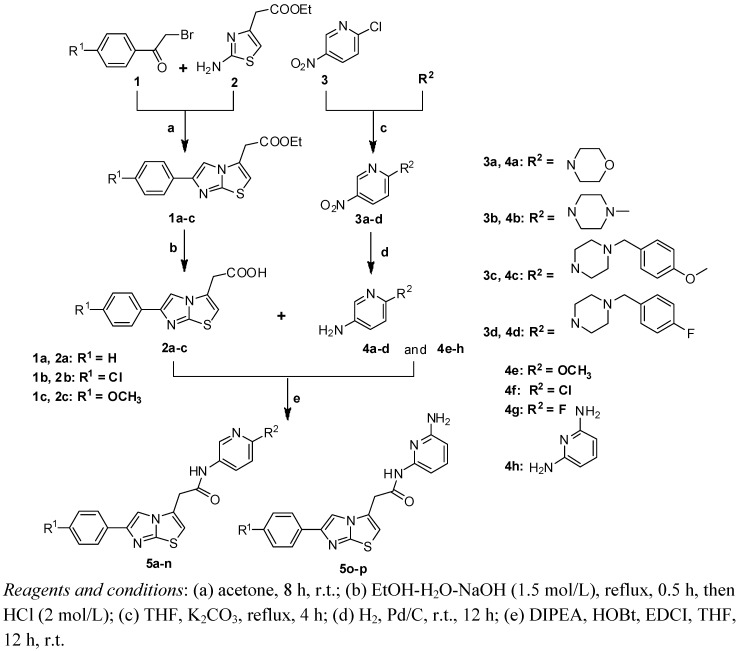
The Synthesis Route of Compounds **5a–p**.

All the agents, unless mentioned otherwise, are commercially available and were directly used without further purification. Synthesis of the intermediates and target compounds was accomplished according to the steps illustrated in Scheme 1. Ethyl 2-(2-aminothiazol-4-yl)acetate (**2**) reacted with substituted or nonsubstituted 2-bromoacetophenones **1** to give the crude products **1a–c** [12], which without further purification were refluxed in EtOH-H_2_O-NaOH (1.5 mol/L), acidified by 2M HCl, to afford **2a–c** in good yields (62%–80%) [24]. Compounds **3a–d** were prepared by the reaction of 2-chloro-5-nitropyridine (**3**) with various R^2^groups in high yield (96%–99%) [25]. Compounds **4a–d** which were obtained by the reduction of **3a–d** [26], along with commercial agents **4e–h**, were coupled with **2a–c** to afford the target compounds **5a–p**.

### 2.2. *In Vitro* Bioactivity

Compound **5a** acquired by the SVM screening model was first evaluated for its inhibitory activity on VEGFR2 kinase and against HepG2 and MDA-MB-231 cell lines. Although **5a** at 20 µM exhibited only a 3.76% inhibitory rate on VEGFR2 kinase, it displayed moderate cytotoxic activity against the HepG2 and MDA-MB-231 cell lines (IC_50_ = 74.2 and 27.1 μM, respectively), which made it a potential hit compound for further investigation. The other 15 compounds were also evaluated against HepG2 and MDA-MB-231 cell lines with sorafenib as the positive control. The IC_50_ value results are summarized in Table 1.

**Table 1:** The substituents and *in vitro* cytotoxicity of synthesized compounds against HepG2 and MDA-MB-231 cell lines. 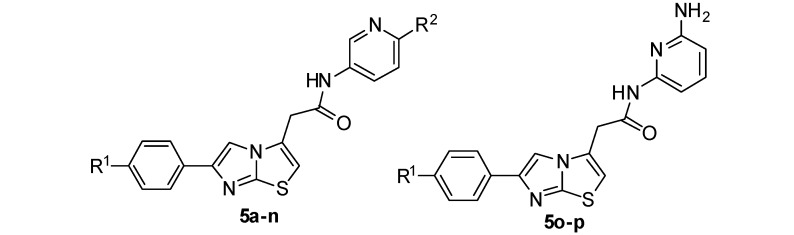

Compound	R^1^	R^2^	IC_50_* (µM)	
			HepG2	MDA-MB-231
**5a**	H	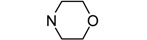	74.2 ± 2.5	27.1 ± 0.4
**5b**	H	OCH_3_	>100	>100
**5c**	Cl	OCH_3_	63.7 ± 0.4	40.1 ± 1.3
**5d**	H	Cl	62.0 ± 3.7	22.8 ± 4.6
**5e**	H	F	>100	79.0 ± 3.8
**5f**	Cl	Cl	50.0 ± 1.4	13.0 ± 0.2
**5g**	Cl	F	53.4 ± 0.5	22.3 ± 1.3
**5h**	OCH_3_	Cl	>100	51.8 ± 0.8
**5i**	H	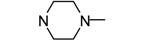	71.5 ± 1.7	>100
**5j**	Cl	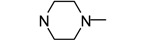	28.2 ± 0.6	>100
**5k**	H	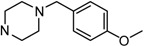	39.4 ± 1.9	6.0 ± 0.7
**5l**	Cl	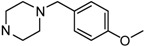	22.6 ± 1.5	1.4 ± 0.1
**5m**	H	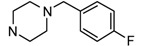	55.2 ± 1.5	19.8 ± 2.2
**5n**	Cl	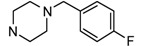	34.7 ± 0.4	12.9 ± 0.2
**5o**	H		>100	62.6 ± 3.7
**5p**	Cl		48.9 ± 1.4	35.1 ± 0.5
**sorafenib**			33.7 ± 1.3	5.2 ± 0.2

* The IC_50_ values were reported as the average of three independent determinations and expressed as the mean ± SD.

As shown in Table 1, some of the modified compounds showed tantamount or better cytotoxicity against either HepG2 or MDA-MB-231 cell line compared with hit compound **5a**. In particular, **5l** exhibited potent activity against MDA-MB-231 cell line, slightly better than sorafenib, with IC_50_ values of 1.4 µM and 5.2 µM, respectively, though it displayed only a little increased inhibition on VEGFR2 kinase (5.72%, inhibitory rate at 20 µM) compared with **5a** (3.76%, inhibitory rate at 20 µM); Compared to the positive control sorafenib, whose activity against the MDA-MB-231 cell line was 7-fold stronger than HepG2, our synthesized compound **5l** demonstrated 16-fold stronger inhibition against MDA-MB-231 than HepG2. Moreover, **5l** showed much lower toxicity towards the HL7702 cell line (normal liver cell) with an IC_50_ value of more than 100 µM, indicating that it may be considered a lead structure for the design of novel useful anticancer agents with low toxicity. Besides **5l**, **5k** with an IC_50_ value of 6.0 µM also displayed good activity which is equivalent to that of sorafenib.

The structure-activity relationships (SARs) suggested that all of compounds with Cl substituents at the R^1^ position displayed better cytotoxic activities than those substituted by H atom or OCH_3_ groups on the same position, including **5c** *vs*. **5b**, **5f** *vs*. **5d** or **5h**, **5g** *vs*. **5e**, **5j** *vs*. **5i**, **5l** *vs*. **5k**, **5n** *vs*. **5m** and **5p** *vs*. **5o**. When R^1^ is H atom, morpholine on R^2^ position replaced by smaller groups, methoxyl or F, decreased the cytotoxicity against both cell lines, but Cl atom showed similar activity to **5a**. Trying 1-methylpiperazine on this position to produce **5j** increased the cytotoxic activity against the HepG2 cell line but considerably decreased cytotoxicity against the MDA-MB-231 cell line, with IC_50_ values of 28.2 μM and >100 μM, respectively. Compounds **5k** and **5m** with further substituted piperazine groups slightly increased the cytotoxic activity, especially **5k** increased much more against MDA-MB-231 cell line. The above observation indicates that pyridine substituted by bulk groups is beneficial for the antitumor activity. Compounds **5o** and **5p** with *N*-(6-aminopyridin-2-yl) moieties displayed lower or similar cytotoxic activity against both cell lines, compared with most of other compounds, which may indicate that the N position on the pyridine ring is important for the cytotoxic activity, or that aminopyridine groups with much larger hydrophilicity hamper the activity. More studies need to be carried out for confirmation of this conjecture.

## 3. Experimental

### 3.1. Materials and Reagents

^1^H-NMR, and ^13^C-NMR spectra were determined on a Bruker ARX-400 400 MHz spectrometer with tetramethylsilane (TMS) as the internal standard and DMSO-*d*_6_ or CDCl_3_ as the solvents (chemical shifts in ppm). Splitting patterns were designated as follows: s: singlet; d: doublet; t: triplet; m: multiplet. Mass spectra were carried out using a Waters Micromass Q-TOF Premier Mass Spectrometer. Melting points were determined in open glass capillaries with a SGW X-4 digital apparatus and were uncorrected. Follow-up of the reactions and checking the homogeneity of the compounds were made by TLC (thin layer chromatography) on silica gel-protected glass plates and the spots were detected by exposure to UV-lamp at *λ*254 and *λ*365. Unless otherwise noted, all solvents and reagents were commercially available and used without further purification.

### 3.2. Chemical Synthesis

#### 3.2.1. General Procedure for Preparation of 2-(6-Phenylimidazo[2,1-*b*]thiazol-3-yl)acetic Acid Derivatives **2a–c**

Ethyl 2-(2-aminothiazol-4-yl) acetate (20 mmol) was dissolved in acetone (50 mL) and treated with the substituted 2-bromoacetophenone (20 mmol, 1 eq.), the mixture was refluxed for 8 h. and then concentrated to 20 mL. The resulting solution was cautiously basified with 15% NH_4_OH to pH 8–9, then poured into CH_2_Cl_2_, separated, washed and concentrated. The crude product was dissolved in EtOH-H_2_O-NaOH (1.5 mol/L) and refluxed for another 0.5 h, then acidified to pH 3–4 with 2M HCl to afford white solid precipitate, following by wash and dry, to afford the desired compounds **2a–c**.

*2-(6-Phenylimidazo[2,1-b]thiazol-3-yl)acetic acid *(**2a**, C_13_H_10_N_2_O_2_S; M.W.: 258.0463). Yield: 79%; mp: 221–223 °C; ^1^H-NMR (DMSO-*d*_6_) *δ*: 8.72 (s, 1H, imidazole H), 7.91 (d, *J *= 7.6 Hz, 2H, Ar-H), 7.55–7.41 (m, 4H, Ar-H and thiazole H), 4.12 (s, 2H, CH_2_); ^13^C-NMR (DMSO-*d*_6_) *δ*: 169.3, 147.4, 139.3, 129.1, 128.9, 128.7, 127.6, 125.0, 114.5, 110.0, 32.2.

*2-(6-(4-Chlorophenyl)imidazo[2,1-b]thiazol-3-yl)acetic acid* (**2b**, C_13_H_9_ClN_2_O_2_S; M.W.: 292.0073). Yield: 80%; mp: 236–238 °C; ^1^H-NMR (DMSO-*d*_6_) *δ*: 8.50 (s, 1H, imidazole H), 7.89 (d, *J *= 8.4 Hz, 2H, Ar-H), 7.52 (d, *J *= 8.4 Hz, 2H, Ar-H), 7.30 (s, 1H, thiazole H), 4.04 (s, 2H, CH_2_); ^13^C-NMR (DMSO-*d*_6_) *δ*: 169.4, 148.2, 141.8, 132.3, 130.6, 128.8, 126.7, 126.5, 112.3, 109.6, 32.6.

*2-(6-(4-Methoxyphenyl)imidazo[2,1-b]thiazol-3-yl)acetic acid* (**2c**, C_14_H_12_N_2_O_3_S; M.W.: 288.0569). Yield: 62%; mp: 231–233 °C; ^1^H-NMR (DMSO-*d*_6_)*δ*: 8.57 (s, 1H, imidazole H), 7.83 (d, *J *= 8.4 Hz, 2H, Ar-H), 7.46 (s, 1H, thiazole H), 7.09 (d, *J *= 8.4 Hz, 2H, Ar-H), 4.10 (s, 2H, CH_2_), 3.82 (s, 3H, CH_3_); ^13^C-NMR (DMSO-*d*_6_) *δ*: 169.2, 159.7, 147.0, 139.7, 127.5, 126.6, 121.3, 114.6, 114.0, 108.7, 55.3, 32.2.

#### 3.2.2. General Procedure for Preparation of Compounds **3a–d**

A mixture of morpholine (100 mmol), 2-chloro-5-nitropyridine (50 mmol), and K_2_CO_3_ (100 mmol) in THF (50 mL) were stirred at 80 °C for 4 h, and then concentrated to 20 mL, poured into water (100 mL), whereby a yellow solid precipitate formed. The precipitate was washed, dried and recrystallized with CH_2_Cl_2_ to afford yellow crystals of **3a**. Compounds **3b–d** were obtained as the same procedure as **3a**.

*4-(5-Nitropyridin-2-yl)morpholine *(**3a**, C_9_H_11_N_3_O_3_; M.W.: 209.0800). Yield: 99%; mp: 141–143 °C; ^1^H-NMR (CDCl_3_) *δ*: 9.04 (d, *J *= 2.8 Hz, 1H, pyridine H), 8.23 (dd, *J *= 2.8, 9.6 Hz, 1H, pyridine H), 6.57 (d, *J *= 9.6 Hz, 1H, pyridine H), 3.82 (t, *J *= 4.8 Hz, 4H, CH_2_), 3.75 (t, *J *= 4.8 Hz, 4H, CH_2_); ^13^C-NMR (CDCl_3_) *δ*: 160.6, 146.3, 135.5, 133.1, 104.5, 66.5, 45.1.

*1-Methyl-4-(5-nitropyridin-2-yl)piperazine *(**3b**, C_10_H_14_N_4_O_2_; M.W.: 222.1117). Yield: 97%; mp: 97–99 °C; ^1^H-NMR (DMSO-*d*_6_) *δ*: 9.02 (d, *J *= 2.4 Hz, 1H, pyridine H), 8.19 (dd, *J *= 2.4, 9.6 Hz, 1H, pyridine H), 6.57 (d, *J *= 9.6 Hz, 1H, pyridine H), 3.79 (t, *J *= 4.8 Hz, 4H, CH_2_), 2.52 (t, *J *= 4.8 Hz, 4H, CH_2_), 2.36 (s, 3H, CH_3_); ^13^C-NMR (DMSO-*d*_6_) *δ*: 160.4, 146.5, 135.1, 133.0, 104.5, 54.6, 46.5, 44.9.

*1-(4-Methoxybenzyl)-4-(5-nitropyridin-2-yl)piperazine *(**3c**, C_17_H_20_N_4_O_3_; M.W.: 328.1535). Yield: 96%; mp: 110–112 °C; ^1^H-NMR (DMSO-*d*_6_) *δ*: 8.94 (d, *J *= 2.8 Hz, 1H, pyridine H), 8.20 (dd, *J *= 2.8, 9.6 Hz, 1H, pyridine H), 7.24 (d, *J *= 8.4 Hz, 2H, Ar-H), 6.92 (d, *J *= 9.6 Hz, 1H, pyridine H), 6.90 (d, *J *= 8.8 Hz, 2H, Ar-H), 3.74 (brs, 7H, CH_2_ and CH_3_), 3.47 (brs, 2H, CH_2_), 2.45 (brs, 4H, CH_2_); ^13^C-NMR (DMSO-*d*_6_) *δ*: 160.0, 158.3, 146.0, 134.1, 132.7, 130.1, 113.5, 105.5, 61.0, 54.9, 51.9, 44.4.

*1-(4-Fluorobenzyl)-4-(5-nitropyridin-2-yl)piperazine* (**3d**, C_16_H_17_FN_4_O_2_; M.W.: 316.1336). Yield: 98%; mp: 99–101 °C; ^1^H-NMR (CDCl_3_) *δ*: 9.00 (d, *J *= 2.4 Hz, 1H, pyridine H), 8.18–8.15 (m, 1H, pyridine H), 7.31 (dd, *J *= 5.2, 8.8 Hz, 2H, Ar-H), 7.03 (d, *J *= 8.8 Hz, 2H, Ar-H), 6.54 (d, *J *= 9.6 Hz, 1H, pyridine H), 3.77 (t, *J *= 5.2 Hz, 4H, CH_2_), 3.52 (s, 2H, CH_2_), 2.53 (t, *J *= 5.2 Hz, 4H, CH_2_); ^13^C-NMR (CDCl_3_) *δ*: 162.2, 160.4, 146.5, 134.9, 133.3, 132.9, 130.6, 115.2, 104.5, 62.0, 52.5, 44.9.

#### 3.2.3. General Procedure for Preparation of Compounds **4a–d**

The mixture of **3a–d** (20 mmol) and Pd/C (20%, 500 mg) in methanol was hydrogenated at atmosphere at r.t. for 12 h, followed by filtration and concentration, to afford crude residue **4a–d**, which was purified by recrystallization (ethanol).

*6-Morpholinopyridin-3-amine* (**4a**, C_9_H_13_N_3_O; M.W.: 179.1059). Yield: 70%; mp: 119–121 °C; ^1^H-NMR (CDCl_3_) *δ*: 7.79 (d, *J *= 2.8 Hz, 1H, pyridine H), 7.00 (dd, *J *= 2.8, 8.8 Hz, 1H, pyridine H), 6.56 (d, *J *= 8.8 Hz, 1H, pyridine H), 3.83 (t, *J *= 4.8 Hz, 4H, CH_2_), 3.33 (t, *J *= 4.8 Hz, 4H, CH_2_), 3.26 (brs, H, NH_2_); ^13^C-NMR (CDCl_3_) *δ*: 154.4, 135.2, 135.1, 126.0, 108.3, 66.8, 47.1.

*6-(4-Methylpiperazin-1-yl)pyridin-3-amine* (**4b**, C_10_H_16_N_4_; M.W.: 192.1375). Yield: 76%; mp: 91–93 °C; ^1^H-NMR (CDCl_3_) *δ*: 7.78 (d, *J *= 2.8 Hz, 1H, pyridine H), 6.98 (dd, *J *= 2.8, 8.8 Hz, 1H, pyridine H), 6.57 (d, *J *= 8.8 Hz, 1H, pyridine H), 3.39 (t, *J *= 4.8 Hz, 4H, CH_2_), 3.29 (brs, 2H, NH_2_), 2.54 (t, *J *= 4.8 Hz, 4H, CH_2_), 2.34 (s, 3H, CH_3_); ^13^C-NMR (CDCl_3_) *δ*: 154.4, 135.2, 134.7, 126.1, 108.5, 55.0, 46.7, 46.2.

*6-(4-(4-Methoxybenzyl)piperazin-1-yl)pyridin-3-amine* (**4c**, C_17_H_22_N_4_O; M.W.: 298.1794). Yield: 74%; mp: 104–106 °C; ^1^H-NMR (CDCl_3_) *δ*: 7.78 (d, *J *= 2.8 Hz, 1H, pyridine H), 7.25 (d, *J *= 8.4 Hz, 2H, Ar-H), 6.96 (dd, *J *= 2.8, 8.8 Hz, 1H, pyridine H), 6.87 (dd, *J *= 2.8, 8.8 Hz, 2H, Ar-H), 6.54 (d, *J *= 8.8 Hz, 1H, pyridine H), 3.84 (brs, 2H, NH_2_), 3.80 (s, 3H, CH_3_), 3.52 (s, 2H, CH_2_), 3.37 (t, *J *= 4.8 Hz, 4H, CH_2_), 2.58 (t, *J *= 4.8 Hz, 4H, CH_2_); ^13^C-NMR (CDCl_3_) *δ*: 158.9, 154.6, 135.4, 134.6, 130.5, 129.7, 126.2, 113.7, 108.4, 62.3, 55.3, 52.7, 46.6.

*6-(4-(4-Fluorobenzyl)piperazin-1-yl)pyridin-3-amine* (**4d**, C_16_H_19_FN_4_; M.W.: 286.1594). Yield: 80%; mp: 94–96 °C; ^1^H-NMR (CDCl_3_) *δ*: 7.76 (d, *J *= 2.8 Hz, 1H, pyridine H), 7.29 (t, *J *= 6.8 Hz, 2H, Ar-H), 6.99 (t, *J *= 8.8 Hz, 2H, Ar-H), 6.92 (dd, *J *= 2.8, 8.8 Hz, 1H, pyridine H), 6.52 (d, *J *= 8.8 Hz, 1H, pyridine H), 3.48 (s, 2H, NH_2_), 3.35 (t, *J *= 4.8 Hz, 6H, CH_2_), 2.53 (t, *J *= 4.8 Hz, 4H, CH_2_); ^13^C-NMR (CDCl_3_) *δ*: 161.9, 154.3, 135.1, 134.8, 133.8, 130.5, 126.0, 115.0, 108.4, 62.2, 52.8, 46.7.

#### 3.2.4. General Procedure for Preparation of Compounds **5a–p**

A solution of the **2a** (1 mmol), **4a** (1 mmol), 1-(3-dimethylaminopropyl)-3-ethylcarbodiimide hydrochloride (EDCI, 1 mmol), *N*-hydroxybenzotrizole (HOBt, 1 mmol), and *N*-diisopropylethylamine (DIPEA, 3 mmol) in anhydrous THF (10 mL) was stirred for 24 h. The reaction was quenched with 1M NaOH (20 mL) and extracted with ethyl acetate (3 × 20 mL), the organic layer was washed with 1M HCl (3 × 20 mL), water (20 mL), dried with Na_2_SO_4_ and evaporated to give compound **5a** as a white solid. Other title compounds **5b–p** were synthesized using the same procedure.

*N-(6-Morpholinopyridin-3-yl)-2-(6-phenylimidazo[2,1-b]thiazol-3-yl)acetamide* (**5a**). Yield: 72%; mp: 212–214 °C; ^1^H-NMR (DMSO-*d*_6_) *δ*: 10.23 (s, 1H, NH), 8.33 (d, *J *= 2.4 Hz, 1H, pyridine H), 8.24 (s, 1H, imidazole H), 7.84–7.81 (m, 3H, Ar-H and pyridine H), 7.39 (t, *J *= 7.6 Hz, 2H, Ar-H), 7.25 (t, *J *= 7.6 Hz, 1H, Ar-H), 7.08 (s, 1H, thiazole H), 6.83 (d, *J *= 9.2 Hz, 1H, pyridine H), 3.99 (s, 2H, CH_2_), 3.69 (t, *J *= 4.8 Hz, 4H), 3.36 (t, *J *= 4.8 Hz, 4H); ^13^C-NMR (DMSO-*d*_6_) *δ*: 165.4, 156.0, 148.5, 145.9, 139.1, 134.2, 130.0, 128.5, 126.9, 126.7, 126.6, 124.6, 109.9, 108.3, 106.8, 65.8, 45.5, 34.9; MS (Q-TOF) *m/z*: 420.1491 [M+H]^+^ (calc. for C_22_H_21_N_5_O_2_S, 419.1416).

*N-(6-Methoxypyridin-3-yl)-2-(6-phenylimidazo[2,1-b]thiazol-3-yl)acetamide* (**5b**). Yield: 76%; mp: 184–186 °C; ^1^H-NMR (DMSO-*d*_6_) *δ*: 10.48 (s, 1H, NH), 8.42 (s, 1H, pyridine H), 8.31 (s, 1H, imidazole H), 7.96 (d, *J *= 1.6 Hz, 1H, Ar-H), 7.94 (s, 1H, Ar-H), 7.86 (d, *J *= 7.6 Hz, 1H, pyridine H), 7.41 (t, *J *= 7.2 Hz, 2H, Ar-H), 7.27 (t, *J *= 7.2 Hz, 1H, Ar-H), 7.14 (s, 1H, thiazole H), 6.83 (d, *J *= 8.8 Hz, 1H, pyridine H), 4.06 (s, 2H, CH_2_), 3.83 (s, 3H, CH_3_); ^13^C-NMR (DMSO-*d*_6_)*δ*: 165.6, 159.8, 148.5, 145.6, 137.8, 33.9, 131.5, 129.7, 128.6, 127.1, 126.6, 124.7, 110.4, 110.1, 108.5, 53.1, 34.9; MS (Q-TOF) *m/z*: 365.1066 [M+H]^+^ (calc. for C_19_H_16_N_4_O_2_S, 364.0994).

*2-(6-(4-Chlorophenyl)imidazo[2,1-b]thiazol-3-yl)-N-(6-methoxypyridin-3-yl)acetamide* (**5c**). Yield: 75%; mp: 247–249 °C; ^1^H-NMR (DMSO-*d*_6_) *δ*: 10.39 (s, 1H, NH), 8.39 (d, *J *= 2.8 Hz, 1H, pyridine H), 8.31 (s, 1H, imidazole H), 7.94 (dd, *J *= 2.8, 8.8 Hz, 1H, pyridine H), 7.87 (d, *J *= 8.8 Hz, 2H, Ar-H), 7.45 (d, *J *= 8.4 Hz, 2H, Ar-H), 7.12 (s, 1H, thiazole H), 6.82 (d, *J *= 8.8 Hz, 1H, pyridine H), 4.03 (s, 2H, CH_2_), 3.83 (s, 3H, CH_3_); ^13^C-NMR (DMSO-*d*_6_)*δ*: 165.6, 159.8, 148.8, 144.8, 137.7, 133.2, 131.5, 131.3, 129.7, 128.6, 126.5, 126.3, 110.4, 110.1, 108.9, 53.1, 34.9; MS (Q-TOF) *m/z*: 399.0683 [M+H]^+^ (calc. for C_19_H_15_ClN_4_O_2_S, 398.0604).

*N-(6-Chloropyridin-3-yl)-2-(6-phenylimidazo[2,1-b]thiazol-3-yl)acetamide *(**5d**). Yield: 70%; mp: 161–163 °C; ^1^H-NMR (DMSO-*d*_6_) *δ*: 11.21 (s, 1H, NH), 8.73 (d, *J *= 4.0 Hz, 1H, imidazole H), 8.70 (d, *J *= 2.8 Hz, 1H, pyridine H), 8.14 (dd, *J *= 2.8, 8.8 Hz, 1H, pyridine H), 7.88 (d, *J *= 7.2 Hz, 2H, Ar-H), 7.53–7.48 (m, 4H, Ar-H and thiazole H and pyridine H), 7.41 (t, *J *= 7.2 Hz, 1H, Ar-H), 4.26 (s, 2H, CH_2_); ^13^C-NMR (DMSO-*d*_6_)*δ*: 166.1, 147.7, 143.8, 140.7, 140.4, 135.1, 129.8, 129.0, 128.6, 127.5, 127.4, 125.0, 124.1, 113.9, 109.9, 34.4; MS (Q-TOF) *m/z*: 369.0573 [M+H]^+^ (calc. for C_18_H_13_ClN_4_OS, 368.0499).

*N-(6-Fluoropyridin-3-yl)-2-(6-phenylimidazo[2,1-b]thiazol-3-yl)acetamide* (**5e**). Yield: 77%; mp: 98–100 °C; ^1^H NMR (DMSO-*d*_6_) *δ*: 10.13 (s, 1H, NH), 8.32 (d, *J *= 1.6 Hz, 1H, imidazole H), 8.22–8.18 (m, 1H, pyridine H), 7.69 (d, *J *= 7.2 Hz, 2H, Ar-H), 7.57 (s, 1H, pyridine H), 7.34 (t, *J *= 7.2 Hz, 2H, Ar-H), 7.26 (dd, *J *= 1.6, 7.2 Hz, 1H, Ar-H), 6.87 (dd, *J *= 2.8, 8.8 Hz, 1H, pyridine H), 6.45 (s, 1H, thiazole H), 3.68 (s, 2H, CH_2_); ^13^C-NMR (DMSO-*d*_6_)*δ*: 165.5, 160.0, 149.7, 147.5, 138.7, 133.5, 133.4, 132.9, 129.0, 128.0, 125.3, 125.2, 110.2, 109.6, 107.3, 36.1; MS (Q-TOF) *m/z*: 353.0876 [M+H]^+^ (calc. for C_18_H_13_FN_4_OS, 352.0794).

*2-(6-(4-Chlorophenyl)imidazo[2,1-b]thiazol-3-yl)-N-(6-chloropyridin-3-yl)acetamide* (**5f**). Yield: 72%; mp: 215–217 °C; ^1^H-NMR (DMSO-*d*_6_) *δ*: 10.73 (s, 1H, NH), 8.62 (d, *J *= 2.0 Hz, 1H, pyridine H), 8.31 (s, 1H, imidazole H), 8.10 (dd, *J *= 2.8, 8.4 Hz, 1H, pyridine H), 7.85 (d, *J *= 8.4 Hz, 2H, Ar-H), 7.50 (d, *J *= 8.4 Hz, 1H, pyridine H), 7.44 (d, *J *= 8.4 Hz, 2H, Ar-H), 7.13 (s, 1H, thiazole H), 4.08 (s, 2H, CH_2_); ^13^C-NMR (DMSO-*d*_6_)*δ*: 166.3, 148.8, 144.8, 143.8, 140.4, 135.1, 133.1, 131.2, 129.9, 128.5, 126.3, 126.1, 124.2, 110.6, 108.9, 35.0; MS (Q-TOF) *m/z*: 403.0188 [M+H]^+^ (calc. for C_18_H_12_Cl_2_N_4_OS, 402.0109).

*2-(6-(4-Chlorophenyl)imidazo[2,1-b]thiazol-3-yl)-N-(6-fluoropyridin-3-yl)acetamide* (**5g**). Yield: 74%; mp: 211–213 °C; ^1^H–NMR (DMSO-*d*_6_) *δ*: 10.66 (s, 1H, NH), 8.45 (brs, 1H, pyridine H), 8.31 (s, 1H, imidazole H), 8.21–8.17 (m, 1H, pyridine H), 7.86 (dd, *J *= 2.0, 6.8 Hz, 2H, Ar-H), 7.45 (dd, *J *= 2.0, 6.8 Hz, 2H, Ar-H), 7.19 (dd, *J *= 3.2, 8.8 Hz, 2H, pyridine H), 7.14 (s, 1H, thiazole H), 4.07 (s, 2H, CH_2_); ^13^C-NMR (DMSO-*d*_6_)*δ*: 166.1, 158.7, 148.8, 144.8, 137.8, 133.8, 133.2, 132.9, 131.3, 128.6, 126.3, 126.2, 110.6, 109.3, 108.9, 34.9; MS (Q-TOF) *m/z*: 387.0480 [M+H]^+^ (calc. for C_18_H_12_ClFN_4_OS, 386.04040.

*N-(6-Chloropyridin-3-yl)-2-(6-(4-methoxyphenyl)imidazo[2,1-b]thiazol-3-yl)acetamide* (**5h**). Yield: 81%; mp: 108–110 °C; ^1^H-NMR (CDCl_3_) *δ*: 10.24 (s, 1H, NH), 8.52 (d, *J *= 2.8 Hz, 1H, pyridine H), 8.16 (dd, *J *= 2.8, 8.8 Hz, 1H, pyridine H), 7.61 (d, *J *= 8.8 Hz, 2H, Ar-H), 7.43 (s, 1H, imidazole H), 7.25 (d, *J *= 7.6 Hz, 1H, pyridine H), 6.89 (d, *J *= 8.8 Hz, 2H, Ar-H), 6.45 (s, 1H, thiazole H), 3.80 (s, 3H, CH_3_), 3.66 (s, 2H, CH_2_); ^13^C-NMR (CDCl_3_)*δ*: 165.6, 159.6, 149.5, 147.5, 146.2, 140.8, 134.3, 130.3, 126.5, 126.1, 125.2, 124.5, 114.4, 109.8, 106.2, 55.4, 36.4; MS (Q-TOF) *m/z*: 399.0683 [M+H]^+^ (calc. for C_19_H_15_ClN_4_O_2_S, 398.0604).

*N-(6-(4-Methylpiperazin-1-yl)pyridin-3-yl)-2-(6-phenylimidazo[2,1-b]thiazol-3-yl)acetamide* (**5i**). Yield: 71%; mp: 132–134 °C; ^1^H-NMR (DMSO-*d*_6_) *δ*: 10.27 (s, 1H, NH), 8.31 (d, *J *= 2.4 Hz, 1H, pyridine H), 8.24 (s, 1H, imidazole H), 7.83 (d, *J *= 7.6 Hz, 2H, Ar-H), 7.79 (dd, *J *= 2.4, 9.2 Hz, 1H, pyridine H), 7.39 (t, *J *= 7.6 Hz, 2H, Ar-H), 7.25 (t, *J *= 7.6 Hz, 1H, Ar-H), 7.08 (s, 1H, thiazole H), 6.82 (d, *J *= 9.2 Hz, 1H, pyridine H), 3.99 (s, 2H, CH_2_), 3.40 (t, *J *= 4.8 Hz, 4H, CH_2_), 2.37 (t, *J *= 4.8 Hz, 4H, CH_2_), 2.20 (s, 3H, CH_3_); ^13^C-NMR (DMSO-*d*_6_) *δ*: 165.3, 155.9, 148.6, 146.0, 139.2, 134.2, 130.1, 128.5, 126.9, 126.6, 126.3, 124.6, 109.9, 108.3, 106.8, 54.3, 45.7, 44.9, 34.9; MS (Q-TOF) *m/z*: 433.1808 [M+H]^+^ (calc. for C_23_H_24_N_6_OS, 432.1732).

*2-(6-(4-Chlorophenyl)imidazo[2,1-b]thiazol-3-yl)-N-(6-(4-methylpiperazin-1-yl)pyridin-3-yl)-acetamide* (**5j**). Yield: 71%; mp: 118–120 °C; ^1^H-NMR (DMSO-*d*_6_) *δ*: 10.22 (s, 1H, NH), 8.31 (d, *J *= 2.4 Hz, 1H, pyridine H), 8.29 (s, 1H, imidazole H), 7.85(d, *J *= 8.4 Hz, 2H, Ar-H), 7.79 (dd, *J *= 2.4, 9.2 Hz, 1H, pyridine H), 7.45 (d, *J *= 8.8 Hz, 2H, Ar-H), 7.10 (s,1H, thiazole H), 6.82 (d, *J *= 9.2 Hz, 1H, pyridine H), 3.98 (s, 2H, CH_2_), 3.41 (t, *J *= 4.8 Hz, 4H, CH_2_), 2.39 (t, *J *= 4.8 Hz, 4H, CH_2_), 2.21(s, 3H, CH_3_); ^13^C-NMR (DMSO-*d*_6_)*δ*: 165.3, 155.9, 148.8, 144.7, 139.2, 133.1, 131.2, 130.0, 128.6, 126.6, 126.3, 126.2, 110.2, 108.8, 106.8, 54.2, 45.7, 44.9, 34.9; MS (Q-TOF) *m/z*: 467.1423 [M+H]^+^ (calc. for C_23_H_23_ClN_6_OS, 466.1343).

*N-(6-(4-(4-Methoxybenzyl)piperazin-1-yl)pyridin-3-yl)-2-(6-phenylimidazo[2,1-b]thiazol-3-yl)acetamide* (**5k**). Yield: 78%; mp: 92–94 °C; ^1^H-NMR (DMSO-*d*_6_) *δ*: 9.26 (s, 1H, NH), 8.19 (d, *J *= 2.4 Hz, 1H, pyridine H), 7.75–7.71 (m, 3H, Ar-H and pyridine H), 7.58 (s, 1H, imidazole H), 7.34 (t, *J *= 7.6 Hz, 2H, Ar-H), 7.24 (t, *J *= 8.4 Hz, 3H, Ar-H), 6.85 (d, *J *= 8.4 Hz, 2H, Ar-H), 6.51 (d, *J *= 9.2 Hz, 1H, pyridine H), 6.42 (s, 1H, thiazole H), 3.79 (s, 3H, CH_3_), 3.60 (s, 2H, CH_2_), 3.45 (s, 2H, CH_2_), 3.43 (t, *J *= 4.8 Hz, 4H, CH_2_), 2.47 (t, *J *= 4.8 Hz, 4H, CH_2_); ^13^C-NMR (DMSO-*d*_6_)*δ*: 165.2, 158.8, 157.2, 149.7, 147.6, 140.6, 133.8, 131.4, 130.3, 129.9, 128.8, 127.7, 125.5, 125.2, 125.0, 113.7, 110.0, 107.1, 106.9, 62.4, 55.3, 52.7, 45.5, 36.2; MS (Q-TOF) *m/z*: 539.2231 [M+H]^+^ (calc. for C_30_H_30_N_6_O_2_S, 538.2151).

*2-(6-(4-Chlorophenyl)imidazo[2,1-b]thiazol-3-yl)-N-(6-(4-(4-methoxybenzyl)piperazin-1-yl)pyridin-3-yl)acetamide* (**5l**). Yield: 72%; mp: 116–118 °C; ^1^H-NMR (DMSO-*d*_6_) *δ*: 8.68 (s, 1H, NH), 8.14 (d, *J *= 2.4 Hz, 1H, pyridine H), 7.71 (dd, *J *= 2.4, 9.2 Hz,1H, pyridine H), 7.66 (s, 1H, imidazole H), 7.63 (d, *J *= 3.6 Hz, 2H, Ar-H), 7.30 (d, *J *= 8.4 Hz, 2H, Ar-H), 7.23 (d, *J *= 8.4 Hz, 2H, Ar-H), 6.86 (d, *J *= 8.4 Hz, 2H, Ar-H), 6.53 (s, 1H, thiazole H), 6.52 (d, *J *= 8.8 Hz, 1H, pyridine H), 3.79 (s, 3H, CH_3_), 3.67 (s, 2H, CH_2_), 3.46 (s, 2H, CH_2_), 3.44 (t, *J *= 4.8 Hz, 4H, CH_2_), 2.48 (t, *J *= 4.8 Hz, 4H, CH_2_); ^13^C-NMR (DMSO-*d*_6_)*δ*: 164.9, 158.9, 157.4, 149.9, 146.8, 140.7, 133.3, 132.4, 131.6, 130.4, 129.9, 128.9, 126.5, 125.3, 124.5, 113.7, 110.4, 107.1, 106.8, 62.5, 55.3, 52.7, 45.5, 36.4; MS (Q-TOF) *m/z*: 573.1841 [M+H]^+^ (calc. for C_30_H_29_ClN_6_O_2_S, 572.1761).

*N-(6-(4-(4-Fluorobenzyl)piperazin-1-yl)pyridin-3-yl)-2-(6-phenylimidazo[2,1-b]thiazol-3-yl)acetamide* (**5m**). Yield: 75%; mp: 80–82 °C; ^1^H-NMR (DMSO-*d*_6_) *δ*: 10.22 (s, 1H, NH), 8.32 (d, *J *= 2.4 Hz, 1H, pyridine H), 8.24 (s, 1H, imidazole H), 7.84 (d, *J *= 7.6 Hz, 2H, Ar-H), 7.80 (dd, *J *= 2.4, 8.8 Hz, 1H, pyridine H), 7.41–7.34 (m, 4H, Ar-H), 7.25 (t, *J *= 7.2 Hz, 1H, Ar-H), 7.15 (t, *J *= 9.2 Hz, 2H, Ar-H), 7.09 (s, 1H, thiazole H), 6.80 (d, *J *= 9.2 Hz, 1H, pyridine H), 3.99 (s, 3H, CH_3_), 3.48 (s, 2H, CH_2_), 3.41 (t, *J *= 4.4 Hz, 4H, CH_2_), 2.43 (t, *J *= 4.4 Hz, 4H, CH_2_); ^13^C-NMR (DMSO-*d*_6_)*δ*: 165.3, 161.2, 155.9, 148.6, 146.0, 139.2, 134.2, 134.1, 130.6, 130.1, 128.5, 126.9, 126.6, 126.3, 124.6, 114.8, 109.9, 108.3, 106.8, 61.1, 52.2, 45.1, 34.9; MS (Q-TOF) *m/z*: 527.2030 [M+H]^+^ (calc. for C_29_H_27_FN_6_OS, 526.1915).

*2-(6-(4-Chlorophenyl)imidazo[2,1-b]thiazol-3-yl)-N-(6-(4-(4-fluorobenzyl)piperazin-1-yl)pyridin-3-yl)acetamide* (**5n**). Yield: 73%; mp: 86–88 °C; ^1^H-NMR (CDCl_3_) *δ*: 9.09 (s, 1H, NH), 8.16 (d, *J *= 2.4 Hz, 1H, pyridine H), 7.69 (dd, *J *= 2.4, 8.8 Hz, 1H, pyridine H), 7.61 (d, *J *= 8.4 Hz, 2H, Ar-H), 7.60 (s, 1H, imidazole H), 7.29–7.26 (m, 4H, Ar-H), 6.99 (t, *J *= 8.8 Hz, 2H, Ar-H), 6.49 (d, *J *= 9.2 Hz, 1H, pyridine H), 6.47 (s, 1H, thiazole H), 3.63 (s, 2H, CH_2_), 3.47 (s, 2H, CH_2_), 3.42 (t, *J *= 4.8 Hz, 4H, CH_2_), 2.46 (t, *J *= 4.8Hz, 4H, CH_2_); ^13^C-NMR (CDCl_3_)*δ*: 165.2, 162.1, 157.2, 149.8, 146.6, 140.6, 133.6, 133.2, 132.3, 131.4, 130.6, 128.9, 126.4, 125.5, 124.9, 115.1, 110.3, 107.3, 106.9, 62.2, 52.7, 45.5, 36.1; MS (Q-TOF) *m/z*: 561.1640 [M+H]^+ ^(calc. for C_29_H_26_ClFN_6_OS, 560.1561).

*N-(6-Aminopyridin-2-yl)-2-(6-phenylimidazo[2,1-b]thiazol-3-yl)acetamide* (**5o**). Yield: 70%; mp: 216–218 °C; ^1^H-NMR (DMSO-*d*_6_) *δ*: 10.30 (s, 1H, NH), 8.22 (s, 1H, imidazole H), 7.83 (d, *J *= 7.6 Hz, 2H, Ar-H), 7.41–7.32 (m, 3H, pyridine H and Ar-H), 7.27–7.19 (m, 2H, pyridine H and Ar-H), 7.08 (s, 1H, thiazole H), 6.20 (d, *J *= 8.0 Hz, 1H, pyridine H), 5.82 (s, 2H, NH_2_), 4.03 (s, 2H, CH_2_); ^13^C-NMR (DMSO-*d*_6_)*δ*: 165.9, 158.5, 150.0,148.6, 145.9, 138.8, 134.2, 128.5, 126.9, 126.6, 124.6, 109.9, 108.3, 103.7, 35.2; MS (Q-TOF) *m/z*: 350.1075 [M+H]^+^ (calc. for C_18_H_15_ClN_5_OS, 349.0997).

*N-(6-Aminopyridin-2-yl)-2-(6-(4-chlorophenyl)imidazo[2,1-b]thiazol-3-yl)acetamide* (**5p**). Yield: 72%; mp: 228–230 °C; ^1^H-NMR (DMSO-*d*_6_) *δ*: 10.29 (s, 1H, NH), 8.27 (s, 1H, imidazole H), 7.84 (d, *J *= 8.4 Hz, 2H, Ar-H), 7.44 (d, *J *= 8.4 Hz, 2H, Ar-H), 7.34 (t, *J *= 8.0 Hz, 1H, pyridine H), 7.18 (brd, *J *= 0.8 Hz, 1H, pyridine H), 7.09 (s, 1H, thiazole H), 6.19 (d, *J *= 8.4 Hz, 1H, pyridine H), 5.81 (s, 2H, NH_2_), 4.02 (s, 2H, CH_2_); ^13^C-NMR (DMSO-*d*_6_)*δ*: 165.9, 158.5, 150.0, 148.8, 144.7, 138.8, 133.1, 131.2, 128.6, 126.6, 126.2, 110.2, 108.8, 103.7, 100.8, 35.2; MS (Q-TOF) *m/z*: 384.0690 [M+H]^+^ (calc. for C_18_H_14_ClN_5_OS, 383.0608).

### 3.3. Biological Methods

#### 3.3.1. *In Vitro* Cytotoxic Assay

The cytotoxic activities of compounds **5a–p** were evaluated with HepG2 (liver cancer cell) and MDA-MB-231(breast cancer cell), as well as the toxicity of **5l** towards HL7702 cell line (normal liver cell) by the MTT (3-(4,5-dimethylthiahiazol-2-y1)-2,5-diphenyltetrazolium bromide) method *in vitro*, with sorafenib as positive control. All the cell lines were purchased from the Type Culture Collection of the Chinese Academy of Sciences, Shanghai, China. Two cancer cell lines were cultured in DMEM (Dulbecco’s modified Eagle medium), 10% fetal bovine serum (FBS), 100 μg/mL penicillin, and 100 μg/mL streptomycin in humidified air at 37 °C, 5% CO_2_. HL7702 cell line cultured in RPMI-1640 (Roswell Park Memorial Institute-1640), 10% FBS, and 100 μg/mL streptomycin in humidified air at 37 °C, 5% CO_2_. The cells were then seeded in 96-well tissue culture plate and treated with the synthesized compounds at different concentrations. 48 h later, 15 μL of MTT solution (5 mg/mL) was added to each well and incubated for another 4 h at 37 °C, 5% CO_2_. The formazan precipitate was dissolved in 100 μL DMSO and the absorbance at 495 nm of each well was measured by Multimode Detector DTX880 (Beckman Coulter). Each assay condition was prepared in triplicate, and the experiments were carried out three times. IC_50_ values were obtained by nonlinear regression (Origin 7.5) and represent the concentration at which cell growth was inhibited by 50% and the SD (standard deviation) were derived.

#### 3.3.2. *In Vitro* VEGFR Inhibitory Activity Assay

The assay was tested by HD Biosciences Co., Ltd. (Shanghai, China). General procedures are as followed: Kinase was incubated with substrate (5-FAM-KKKKEEIYFFF-CONH_2_), compounds and ATP (adenosine triphosphate) in a final buffer of 25 mM 4-(2-hydroxyethyl)-1-piperazine-ethanesulfonic acid (HEPES, pH 7.4), 10 mM MgCl_2_, 0.01% Triton X-100, 100 µg/mL albumin from bovine serum (BSA), 2.5 mM DL-dithiothreitol (DTT) in 384-well plate with the total volume of 10 µL. The assay plate was incubated at 30 °C for 1 h and stopped with the addition of equal volume of kinase-glo plus reagent. The luminescence was read at envision. The signal was correlated with the amount of ATP present in the reaction and was inversely correlated with the kinase activity [27].

## 4. Conclusions

In summary, based on the virtual screening hit compound **5a**, we designed and synthesized a series of novel compounds possessing an imidazo[2,1-*b*]thiazole scaffold, and evaluated them for their anticancer activity. The results showed that some of the synthesized compounds displayed better activity than **5a**, against either the HepG2 or MDA-MB-231 cell lines. Of these compounds, **5l** with lower inhibition on VEGFR2 kinase was a potent, selective inhibitor against the MDA-MB-231 cell line compared with the HepG2 cell line. This study may provide valuable information for further design of imidazo[2,1-*b*]thiazole derivatives as potential antitumor agents which could specifically target VEGFR kinase.
